# Profiles of Long Non-Coding RNAs and mRNA Expression in Human Macrophages Regulated by Interleukin-27

**DOI:** 10.3390/ijms20246207

**Published:** 2019-12-09

**Authors:** Xiaojun Hu, Suranjana Goswami, Ju Qiu, Qian Chen, Sylvain Laverdure, Brad T. Sherman, Tomozumi Imamichi

**Affiliations:** Laboratory of Human Retrovirology and Immunoinformatics, Frederick National Laboratory for Cancer Research, Frederick, MD 21702, USA; xiaojun.hu@usda.gov (X.H.); suranjana.goswami@nih.gov (S.G.); ju.qiu@nih.gov (J.Q.); chenq3@mail.nih.gov (Q.C.); sylvain.laverdure@nih.gov (S.L.); bsherman@mail.nih.gov (B.T.S.)

**Keywords:** lncRNA, IL-27, macrophage, RNA-Seq

## Abstract

Macrophages play an essential role in the immune system. Recent studies have shown that long non-coding RNAs (lncRNAs) can regulate genes encoding products involved in the immune response. Interleukin (IL)-27 is a member of the IL-6/IL-12 family of cytokines with broad anti-viral effects that inhibits human immunodeficiency virus (HIV) type-1 and herpes simplex virus (HSV). However, little is known about the role of lncRNAs in macrophages affected by IL-27. Therefore, we investigated the expression profiles of mRNA and lncRNA in human monocyte-derived macrophages (MDMs) regulated by IL-27. Monocytes were differentiated in the presence of macrophage-colony stimulatory factor (M-CSF)- or human AB serum with or without IL-27, and these cells were the subject for the profile analysis using RNA-Seq. We identified 146 lncRNAs (including 88 novel ones) and 434 coding genes were differentially regulated by IL-27 in both M-CSF- and AB serum-induced macrophages. Using weighted gene co-expression network analysis, we obtained four modules. The immune system, cell cycle, and regulation of complement cascade pathways were enriched in different modules. The network of mRNAs and lncRNAs in the pathways suggest that lncRNAs might regulate immune activity in macrophages. This study provides potential insight into the roles of lncRNA in macrophages regulated by IL-27.

## 1. Introduction

Long non-coding RNAs (lncRNAs) are a large group of non-coding transcripts that are more than 200 nucleotides in length [1,2]. LncRNAs are extensively reported to be involved in gene regulation and cellular processes through a diversity of mechanisms such as stability of transcripts and enhancement/suppression of transcription [1,3]. A large number of studies have indicated that dysfunction of lncRNAs is highly associated with a wide variety of diseases (i.e., Alzheimer’s disease, cancer, cardiovascular disease, diabetes, and neurodegeneration disease) [4,5,6,7]. The LncRNADisease database v2.0 documents more than 200k lncRNA-disease associations including 529 diseases and 19,166 lncRNAs [8]. Understanding the role of lncRNAs in the pathogenesis of the disease is a growing and intriguing area of research.

Macrophages are important cells of the immune system. They have a central role in host defense, wound healing, and immune regulation [9,10]. In addition to phagocytosis of the immune complex, cellular debris, and pathogens, they play a pivotal role as antigen-presenting cells (APCs), which trigger antibody responses by the presentation of pathogen derived peptides in conjunction with the major histocompatibility complex (MHC)-II molecules on the cell surface, to CD4+ T cells and initiate inflammation by releasing cytokines to activate other cells [10,11], and macrophage differentiation and activation can be regulated by lncRNAs [12,13,14].

Cytokines are a large group of small soluble proteins that play an important role in the regulation of cell signaling. They are crucial for a wide variety of physiological processes including the regulation of immune and inflammatory responses [15]. LncRNAs can control cytokine production and cytokines, in turn, can regulate lncRNA expression. This interplay between cytokines and lncRNAs is essential in the regulation of the immune system [16]. Understanding the molecular mechanism of their interaction is a daunting challenge.

Interleukin (IL)-27, is a relatively recently discovered heterodimeric type I cytokine in the IL-6/IL-12 cytokine family, composed of IL-27 p28 and Epstein-Barr virus-induced gene 3 (EBI3) subunits [17]. It engages a receptor formed by gp130 and IL-27Rα to activate the Janus kinase/signal transducer and activator of transcription (JAK/STAT) and mitogen activated protein kinase (MAPK) signaling pathways [18]. IL-27 is mainly induced by a number of Toll like receptor (TLR) agonists on monocytes, macrophages, and dendritic cells [19,20]. It displays a pro- or anti-inflammatory dual role in immune regulation [21,22] and also regulates reactive oxygen species [23]. IL-27 can also suppress virus infection [24,25], thus it is considered as a potential therapeutic target in cancer immunotherapy, autoimmune, and infectious diseases [22,26,27,28]. 

IL-27 has previously been shown to inhibit various virus infections such as human immunodeficiency virus (HIV) type-1, HIV-type-2, hepatitis B virus (HBV), herpes simplex virus type 1 (HSV-1), and influenza A [25,29,30,31,32,33]. We have reported that IL-27 induces HIV-1 inhibition in CD4+ T cells, macrophages, and dendritic cells in an interferon (IFN)-independent manner [25,29,34]. We recently reported that microRNA (miRNA) expression in macrophage and dendritic cells can be regulated by IL-27 [35]. We hypothesized that lncRNAs play a role in IL-27 immune networks.

To address our hypotheses, we performed an RNA-Seq analysis using macrophages treated with IL-27. Our results showed that the gene expression profile of lncRNAs and mRNAs were modulated by IL-27. Some immune-related pathways and functions were involved. More importantly, we identified some novel lncRNAs and confirmed their expression by RT-qPCR.

## 2. Results

### 2.1. Identification of Putative Long Noncoding RNA (lncRNA) Transcripts

A total of ten high-throughput RNA-Seq libraries were generated from two experiments, IMAC (IL-27-treated M-CSF-induced macrophages) and ABI (IL-27-treated AB serum-induced macrophages) experiments. In the IMAC experiment, monocytes were differentiated into macrophages with M-CSF in the absence (mMac) or presence of IL-27 (iMac). In the ABI experiment, monocytes were differentiated to macrophages using human AB-Serum in the absence (Ab) or presence of IL-27 (Abi). The paired-wise comparison of different samples showed that there was a good reproducibility in this study (Figure S1). A total of 1.2 billion reads were mapped to the human genome reference build (hg38) with >93% mapping rate for all libraries (Table S1). A total of 170,716 transcripts were assembled by StringTie (v1.3.5). Among them, 22,767 transcripts (length >200 bp) were not annotated to the Ensembl human gene annotation release 93 (GRCh38.93). A computational pipeline (Figure S2) adapted from previous studies [36,37,38] was implemented to screen novel candidate lncRNAs from unannotated transcripts. The protein coding ability of these transcripts was assessed using the Coding-Potential Assessment Tool (CPAT v1.2.3) and Coding-Non-Coding Index (CNCI v2). A total of 4843 non-coding candidate transcripts were retained with default cut-off thresholds <0.364 (CPAT) or <0 (CNCI). Additionally, transcripts with class codes ‘u’ (intergenic), ‘x’ (antisense), and ‘i’ (intronic) from GffCompare (v0.10.4) after comparing to coding proteins were kept. Furthermore, 247 candidates in the GENCODE (v29) lncRNA, NONCODE (v5.0), and LNCipedia (v5.2) databases were filtered with class code ‘=’. To reduce false discovery caused by the mapping bias, all reads were remapped to all annotated transcripts plus candidates using the lightweight mapping tool Salmon (v0.11.4). A total of 318 transcripts found only in one sample or not in the Salmon mapping were removed. Finally, 2691 candidate lncRNA transcripts were obtained (Table S2). Among them, 53% were from intergenic regions, 27% from intronic regions, and 20% were antisense to protein-coding loci.

### 2.2. Expression Profiles of lncRNAs

**Figure 1:**
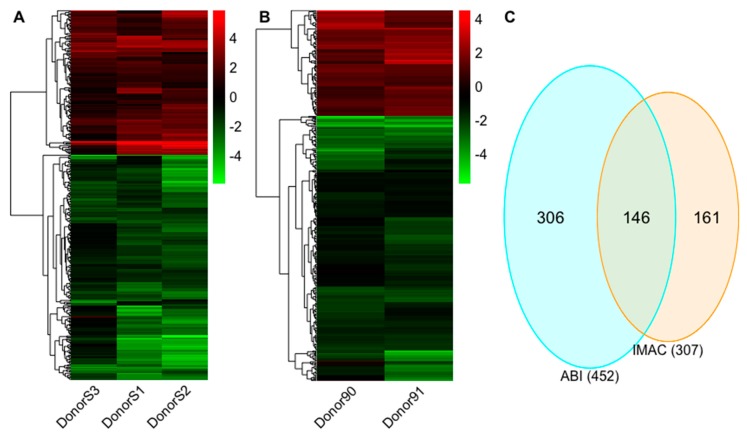
The differential expression profiles for Interleukin (IL)-27 regulated long non-coding RNAs (lncRNAs). (**A**) The hierarchical clustering of differentially regulated lncRNAs for iMac vs. mMac in the IMAC experiment. (**B**) The hierarchical clustering of differentially regulated lncRNAs for Abi vs. Ab in the ABI experiment. (**C**) Venn diagram indicates the number of overlapping and non-overlapping differential lncRNAs in the IMAC and ABI experiments. Values in the heatmap indicate the fold change for each donor. The color scale shown at the right illustrates the relative expression level of the indicated lncRNA in each sample: green denotes downregulated (log_2_ fold change <0) and red denotes upregulated (log_2_ fold change >0).

To determine the differential regulation profile of lncRNAs in response to IL-27 in macrophages, 29,566 lncRNA transcripts from GENCODE (v29) and 3251 candidate lncRNA transcripts (including 560 in NONCODE (v5.0) or LNCipedia (v5.2)) were used as a reference transcriptome for Salmon (v0.11.4) to quantify the samples. The tximport (v1.10.1) package in R (v3.5.3) was used to summarize transcript-level abundance to gene-level counts since it is currently more accurate to analyze differential regulation at the gene-level [39]. To identify the significantly differentially expressed lncRNA genes, a false discovery rate (FDR) <0.05 and ≥two-fold expression change were used as selection criteria for the edgeR (v3.24.3) results. In the IMAC experiment, 120 lncRNAs were upregulated and 187 lncRNAs were downregulated by IL-27 (Figure 1A and Figure S3B). In the ABI experiment, 129 lncRNAs were induced and 323 lncRNAs were downregulated by IL-27 (Figure 1B and Figure S3D). A total of 146 significant differentially expressed lncRNAs overlapped in the two experiments (Figure 1C). Among them, 58 lncRNAs were annotated in GENCODE (v29) (Table S3) while 88 lncRNAs were novel candidates (Table S4). The number of lncRNAs differentially expressed in the subgroups [iMac vs. mMac, Abi vs. Ab] is listed in Table 1. Of note, most of the novel candidates (50 out of 88) were different isoforms of known lncRNAs. Since lncRNAs are often cell type-specific, the determination of whether or not these are macrophage-specific isoforms is of interest for further study.

### 2.3. Expression Profiles of mRNAs

**Figure 2:**
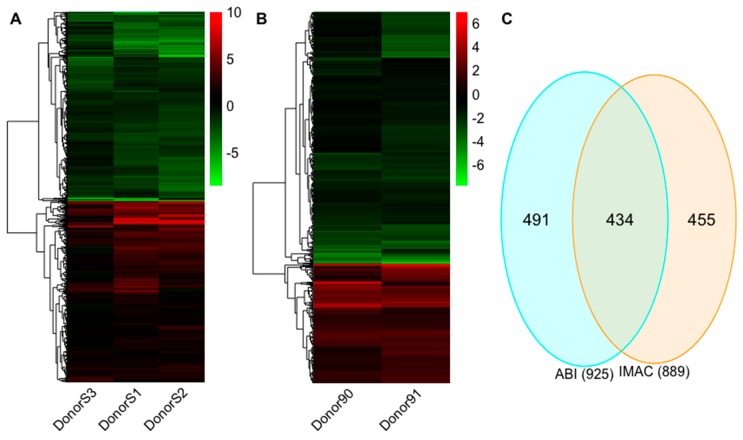
The differential expression profiles for Interleukin-27 (IL-27 regulated mRNAs. (**A**) The hierarchical clustering of differentially regulated mRNAs for iMac vs. mMac in the IMAC experiment. (**B**) The hierarchical clustering of differentially regulated mRNAs for Abi vs. Ab in the ABI experiment. (**C**) Venn diagram indicates the number of overlapping and non-overlapping differential mRNAs in the IMAC and ABI experiments. Values in the heatmap are the fold change for each donor. The color scale shown at the right illustrates the relative expression level of the indicated mRNA in each sample: green denotes downregulated (log_2_ fold change <0) and red denotes upregulated (log_2_ fold change >0).

From the analysis of the IMAC experiment, 434 mRNAs were upregulated, and 455 mRNAs were downregulated by IL-27 (Figure 2A and Figure S3A). In the ABI experiment, 298 mRNAs were upregulated and 627 mRNAs were downregulated by IL-27 (Figure 2B and Figure S3C). A total of 434 significantly differentially regulated mRNAs overlapped in the two experiments (Figure 2C and Table S5).

### 2.4. Enrichment Analysis of Differential mRNAs

Kyoto Encyclopedia of Genes and Genomes (KEGG) and Gene Ontology (GO) enrichment analysis were performed using DAVID (v6.8) [40] based on the 434 significantly differentially expressed mRNAs that overlapped between the IMAC and ABI experiments. These mRNAs were significantly enriched for 18 KEGG pathways (Table 2). Most of them were immune-related pathways such as staphylococcus aureus infection (KEGG: hsa05150), intestinal immune network for IgA production (KEGG: hsa04672), antigen processing and presentation (KEGG: hsa04612), and autoimmune thyroid disease (KEGG: hsa05320). They were also enriched in the cell cycle pathway (KEGG: hsa04110).

There were nine GO biological process (GO-BP), nine GO cellular component (GO-CC), and two GO molecular function (GO-MF) terms significantly enriched (Table 2). In the GO-BP domain, the functions were mainly involved in immune response (GO:0006955), inflammatory response (GO:0006954), antigen processing and presentation (GO:0019882), and cell division (GO:0051301). This result was similar to the KEGG analysis. In the GO-MF domain, peptide antigen binding (GO:0042605) and MHC class II receptor activity (GO:0032395) were associated with differentially expressed genes. In the GO-CC domain, the top GO terms were transport vesicle membrane (GO:0030658) and MHC class II protein complex (GO:0042613).

### 2.5. Co-Expression Network Construction and Module Analysis

The expression of mRNA can be regulated by lncRNAs. Therefore, the expression patterns of lncRNAs and their target mRNAs could be correlated. The R package in the weighted gene correlation network analysis (WGCNA v1.67) is a well-known network analysis tool to identify groups of highly correlated genes. In this study, WGCNA was used to detect the co-expression patterns among the 434 differentially expressed mRNAs and 146 lncRNAs that overlapped in the IMAC and ABI experiments. Highly correlated genes were clustered into four color modules: brown, yellow, turquoise, and blue (Figure S4). The number of differentially expressed mRNAs/lncRNAs in each module were: 81 mRNAs and 19 lncRNAs in brown, 80 mRNAs and 7 lncRNAs in yellow, 121 mRNAs and 52 lncRNAs in blue, and 104 mRNAs and 55 lncRNAs in turquoise. The genes in the same module, which are strongly connected to other genes, may have similar biological functions.

To find the module associated functions, mRNAs in each module were analyzed to detect enriched pathways or GO terms using STRING (v11.0). Table 3 shows a summary of the most significant Reactome/KEGG pathways and GO biological process (GO-BP) terms associated with these modules. The biological functions were enriched in different modules. The pathways in the brown and turquoise modules were activated in the immune system such as defense response, immune response, and inflammatory response. The yellow and blue modules mainly participated in cell cycle regulation and complement activation, respectively.

**Figure 3:**
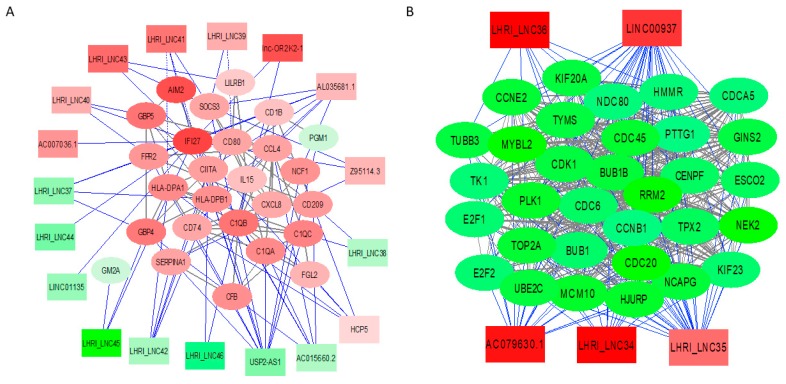
Visualization of lncRNAs and mRNAs in enriched pathways. (**A**) Reactome pathway: immune system in brown module. (**B**) Reactome pathway: cell cycle in the yellow module. Rectangle nodes represent lncRNAs and ellipse nodes represent mRNAs. Solid blue lines denote connections between lncRNAs and their target mRNAs predicted by LncTar. Dashed blue lines denote lncRNA targeting neighbor mRNA genes retrieved by Bedtools. Solid grey lines denote protein–protein interactions determined by STRING. Red color denotes upregulated genes. Green color denotes downregulated genes.

To further explore lncRNA roles, the top two Reactome pathways of immune system (Reactome: HSA-168256) and cell cycle (Reactome: HSA-1640170) were chosen. Their connections to other coding genes in a network were constructed by Cytoscape (v3.7.1). LncRNAs targeting neighbor genes (100k flanking region) were retrieved using Bedtools (v2.25.0). Possible lncRNA binding genes were predicted by LncTar. Figure 3A shows that 26 mRNAs and 20 possible lncRNAs are involved in the immune system pathway. USP2-AS1 had 11 targets and HCP5 (HLA Complex P5) had five targets (CD74, FGL2, HLA-DPA1, IFI27, NCF1). Novel lncRNA LHRI_LNC42 (MK280501) (Table S2) had five targets (CD74, CIITA, HLA-DPA1, HLA-DPB1, and SERPINA1). AC015660.2 and AL035681.1 had six and seven targets, respectively. The other lncRNAs had less than four targets. Figure 3B shows that 32 mRNAs and five possible lncRNAs are known to be involved in the regulation of the cell cycle. Of interest, all mRNAs were downregulated and all lncRNAs were upregulated following IL-27 treatment in this pathway. Each lncRNA had many targets including LINC00937, which had 30 targets and LHRI_LNC35 (MN298422), which had 28 targets. Macrophages are terminally differentiated monocytic phagocytes and do not have a cell cycle, therefore, these results suggest that the five possible lncRNAs may be involved in the regulation of the cell function (e.g., activation of macrophages rather than cell cycle).

### 2.6. Confirmation of the Selected lncRNAs

**Figure 4:**
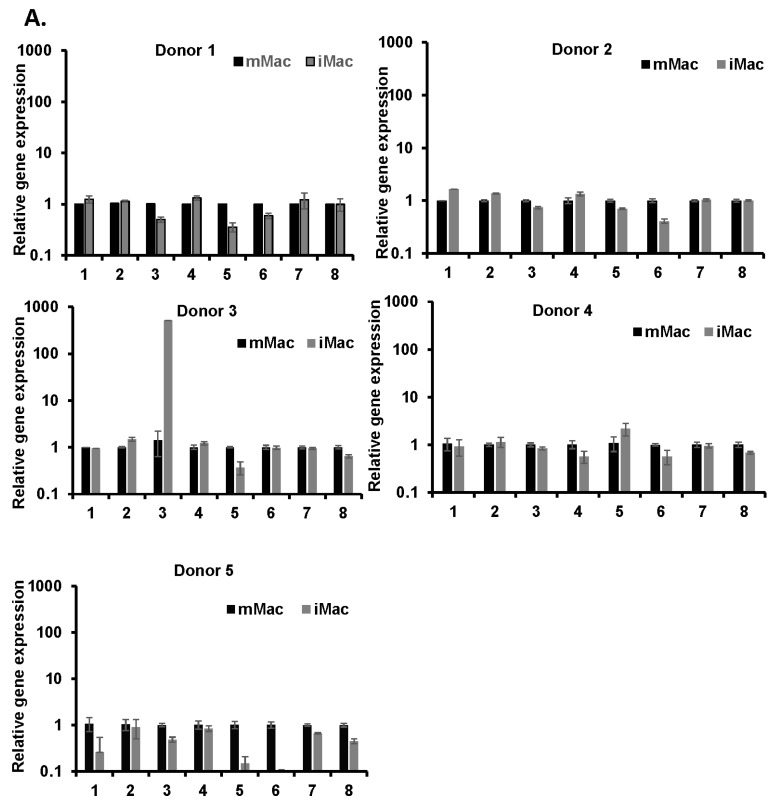

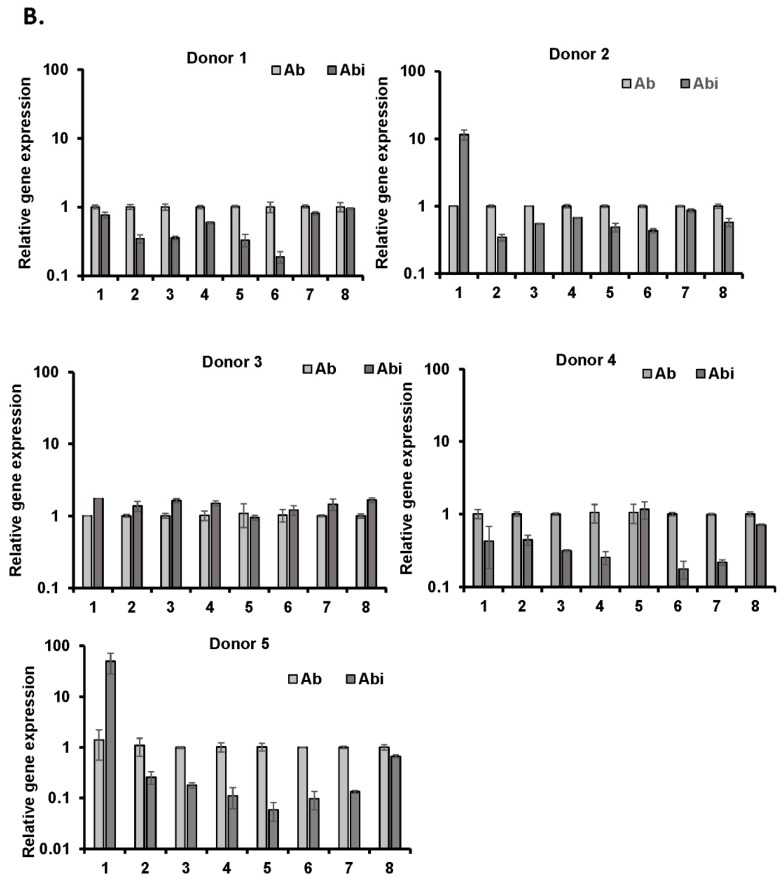
Validation of selected novel lncRNAs by qRT-PCR. (**A**) iMac vs. mMac. (**B**). Abi vs. Ab. Data are expressed as the mean ± SD (*n* = 3). Data were normalized to the house keeping gene GAPDH. The X-axis is LHRI_LNC-1, 2, 3, 4, 5, 6, 7, 8; and the Y-axis is relative gene expression.

Eight novel differentially expressed lncRNAs in both the IMAC and ABI experiments were selected for further validation by qRT-PCR using different donors from RNA-Seq. For the IMAC samples, LHRI_LNC-3 (MK280569) and -5 (MK280118) were similar to the RNA-Seq results (Figure 4A). LHRI_LNC-6 (MN298516) and -8 (MN298210) had the same trend as the RNA-Seq results. LHRI_LNC-1, -2, -4, and -7 (MK280176, MK280257, MK280027, MK280165) showed an opposite trend when compared to the RNA-Seq results. For the ABI samples, LHRI_LNC-2, -3, -4, -5, and -6 (MK280257, MK280569, MK280027, MK280118, MN298516) had the same trend as the RNA-Seq results, but were not significant. For the mMac and iMac comparison, one donor showed a high expression of LHRI_LNC-3 in iMac, whereas they were not upregulated in the other four donors. None of the expression patterns for mMac or Ab were consistent, reinstating the fact that there is donor dependency.

### 2.7. Biological Function Assay

We have previously found that IL-27 inhibits HIV in macrophages and T cells [25,41,42] and as shown in this study, IL-27 modulates lncRNA profiles. To assess the role of several novel lncRNAs as a pilot experiment, we randomly chose four lncRNAs, LHRI_LNC-2, 3, 5, and 6, and made an expression vector. HEK293T cells were transfected with different concentrations (10, 100, and 1000 ng) of each plasmid, followed by HIVluc infection. HIV infection was monitored by luciferase activity as described in the materials and methods. Our results indicated that the selected lncRNAs had no significant impact on HIV infection (Figure S5).

## 3. Discussion

In the present study, we analyzed the mRNA (coding gene) and lncRNA expression profiles and functional networks of IL-27 treated macrophages. We identified 434 protein-coding genes and 146 lncRNAs (including 88 unknown ones) with differential expression in both the ABI and IMAC experiments. Both the KEGG pathway and GO-term enrichment analysis indicated that some cellular functional pathways contribute to IL-27 functions such as immune response, inflammatory response, antigen processing and presentation, and cell cycle. Further co-expression network analysis revealed that some lncRNAs may be involved in the biological functions through their target genes. Therefore, our results provide a comprehensive profile of the lncRNA and mRNA expression in the macrophage regulated by IL-27 and lay a foundation for the understanding of the IL-27 immunoregulatory mechanisms.

Macrophages are one of the antigen-presenting cells in the immune system. They modulate many pathophysiological pathways and are activated by pathogens. The role of lncRNAs in macrophage activation has been revealed by several studies [14,43]. Scacalossi et al. recently reviewed the role of lncRNA in macrophage function such as in macrophage polarization, and innate and adaptive immune functions [13]. Gupta et al. analyzed lncRNAs in bovine macrophages and suggested that lncRNAs could respond to para-tuberculosis infection [44]. Our results showed that lncRNAs regulated by IL-27 in human macrophages were mainly involved in immune response and cell cycle pathways. Vadiveloo reported that cell cycle proteins play a role in macrophage activation [45].

We previously reported that IL-27 inhibits HIV in macrophages [29]. Other researchers have also reported that IL-27 is able to suppress HBV, HCV, HIV-1, HIV-2, HSV, and influenza A [30,31,32,33,34] in various cell types. In this study, KEGG analysis indicated that the differential profile of the expressed gene was enriched in immune diseases such as staphylococcus aureus infection, autoimmune thyroid disease, inflammatory bowel disease (IBD), and graft-versus-host disease. It has been reported that IL-27 is elevated in patients with autoimmune/inflammatory diseases such as Crohn’s disease, rheumatoid arthritis, multiple sclerosis, psoriasis, and aplastic anemia [46]. Mutations in IL-27 could be a causal effect for some autoimmune diseases such as IBD, chronic obstructive pulmonary disease, and asthma [46]. The Gene Ontology (GO) knowledgebase analysis demonstrated that differentially expressed genes were enriched in immune related GO terms such as interferon-gamma (IFN-γ)-mediated signaling pathway, T cell co-stimulation, antigen processing and presentation, and the major histocompatibility complex (MHC) class II protein complex. Rajaiah et al. reported that IL-27 and IFN-γ are involved in the regulation of autoimmune arthritis [47]. Therefore, the data shown here suggest that IL-27 regulates some immune genes to defend against diseases or pathogen infection.

Although it is reported that IL-27 regulates the expression of some coding genes [41], whether or not it can regulate lncRNAs is still unclear. In this study, our data demonstrated that IL-27 not only regulates the coding genes, but also lncRNAs in macrophages. It is well documented that lncRNAs can target neighboring coding genes [48] or bind to RNA-binding proteins to regulate the activities of some genes [49]. To infer whether or not the lncRNAs were involved in IL-27 pathways, we clustered lncRNAs and mRNAs together by their co-expression relationship, and further identified lncRNAs targeting mRNAs in its neighboring location (100 kb flanking region) and potential binding prediction in a pathway of a cluster (module). Reactome (an online database of biological pathways) analysis revealed that in the immune system pathway, 26 coding genes were differentially expressed, and 18 lncRNAs may be involved in the regulation. In the cell cycle pathway, 32 coding genes were differentially expressed, and five lncRNAs may be related to gene induction. Therefore, our results indicate that IL-27 plays an important role in the expression of not only coding genes, but also lncRNAs in the pathways. It is reported that the regulation of genes by lncRNAs might be critical for the immune system [50]. The human leukocyte antigen (HLA) class I histocompatibility antigen protein P5 (HCP5), which localizes within the MHC class I region and is an endogenous retroviral element, was upregulated by IL-27 in our current study. It was reported that HCP5 has homology to the HIV pol gene and polymorphisms in this lncRNA were correlated with a lower HIV-1 viral set point, thus HCP5 could help the immune system reduce HIV viral replication or disease progression [51,52].

LINC00937 was significantly upregulated by IL-27 (Table S3). A result from the Reactome analysis indicated that the lncRNA may regulate 30 differential genes associated with the cell cycle pathway. Xu et al. recently reported that LINC00937 is strongly connected with the prognosis of cutaneous melanoma (CM) [53]. As shown in Figure 3, there are many novel lncRNAs involved in the IL-27 regulated networks, therefore, future studies would be needed to reveal the functions of these lncRNAs.

The expression of lncRNAs is highly cell-type specific [54]. Hu et al. reported that lncRNA expression patterns are related to the T cell subset during T cell differentiation [55]. In this study, 2691 novel lncRNA transcripts were discovered in macrophages. However, 79% of them had other lncRNAs nearby (from GENCODE, NONCODE, and LNCipedia). They are usually different isoforms of a gene. Coding gene alternative splicing is regulated in a cell-type- and developmental-stage-specific manner [56]. Therefore, our data suggest that some lncRNA isoforms might be macrophage specific. Deveson et al. demonstrated that noncoding isoforms generated by alternative splicing could be infinite [57].

LncRNAs regulate gene expression through a variety of mechanisms including lncRNA-mediated inter-chromosomal interaction, guide, or decoys of transcription factor (reviewed in [58,59,60]). LncRNAs regulate their neighboring genes (*cis*) or distal genes (*trans*) [61]. Some lncRNAs play a critical role in chromatin organization [58,62]. They can bind to chromatin-modifying proteins and recruit protein complexes to specific sites to remodel chromatin states and regulate gene expression. XIST (X inactive-specific transcript) and HOTAIR (Hox transcript antisense intergenic RNA) are two interesting lncRNAs for the chromatin remodeling study [63,64,65]. Some lncRNAs are epigenetically regulated through histone modifications like H3K4me3, H3K36me3, and H3K27ac [66,67]. In our study, 74.3% novel lncRNAs were associated with H3K36me3 (a mark of actively transcribed gene bodies), 30.6% with H3K27ac (used to define enhancers), and 27.4% with H3K4me3 (a mark of active promoters) (see details in Table S2). Some lncRNAs can modulate viral infection in an interferon-dependent, -independent manner or other mechanisms (review in [68,69]). Zhang (2013) reported that NEAT1 (Nuclear paraspeckle assembly transcript 1) was involved in the replication of HIV-1 [70]. We tested four novel lnRNAs for an anti-HIV function. Although they did not impact on HIV replication, other functions may be discovered in the future.

In the current study, there were some discrepancies between RNA-Seq and RT-qPCR results. These differences may be caused by several factors. First, in this study, due to a lack of sufficient RNA for performing RT-qPCR of all probes, RNA for RT-qPCR was obtained from different donors for RNA-Seq. As we have previously reported that different donor cells demonstrate a different intensity in the biological response to IL-27 [29], this suggests that the expression of a lncRNA(s) in response to IL-27 may be changed in a donor-dependent manner, thus, as shown in Figure 4A,B, the expression profiles of lncRNAs differed among the donors and this difference may affect the discrepancies between RNA-Seq and RT-qPCR. Second, it may be caused by a bias in the RT-qPCR experiment. Except for its probe-bias based on what region of the cDNA is amplified, the probe affinity/specificity also influences the experimental results, because it affects the efficiency and efficacy of cDNA synthesis. Third, it may be caused by different quantification methods in the analysis of both assays. RT-qPCR results were normalized relative values against an internal control gene product using the delta–delta Ct method, and then the difference of a gene expressed in IL-27-treated cells compared to the control cells was calculated as a relative fold-change. In RNA-Seq analysis, the gene expression level was compared using normalized read count and the fold change was a ratio of read count between the control and IL-27 treated cells. When the read counts are low, the fold change value can be disproportionately affected by measurement noise. Most novel lncRNAs were expressed at a low level as their read counts were small numbers. This bias should be a concern in the lncRNA RNA-Seq study. Fourth, the target of the designed RT-qPCR probe may affect the result. If the probe targets a common region of multiple transcripts of a gene including isoforms, a result from the RT-qPCR using the probes may reflect the gene-level expression. In contrast, if the probe targets the unique region of a transcript, the result may correspond to the transcript-level of the unique region. Significant changes may be observed at the gene-level, but not at the transcript-level.

In summary, our current study shows that IL-27 treatment affects lncRNA and mRNA expression profiles in human primary macrophages. Several GO-term and KEGG/Reactome pathways have been identified. Functional networks suggest that lncRNA might be involved in the immune system and cell cycle pathways through their regulated genes. IL-27 can stimulate many immune cells other than macrophages, thus similar studies of the expression profile of coding genes and lncRNAs in other cell types may provide new insights into IL-27-mediated cellular function.

## 4. Materials and Methods

### 4.1. Cell Purification and Culture

CD14+ monocytes were purified from peripheral blood mononuclear cells (PBMCs) of healthy donors using CD14 MicroBeads (Miltenyi Biotec, Auburn, CA, USA), according to the manufacturer’s instructions as previously described [25]. Approval for these studies was granted by the National Institute of Allergy and Infectious Diseases Institutional (NIAID) Review Board. Participants provided informed written consent before blood was drawn. To generate M-CSF-induced macrophages (mMac) or AB macrophages (Ab), the isolated CD14+ monocytes were cultured for seven days in the presence of 25 ng/mL M-CSF (R&D Systems, Minneapolis, MN, USA) in macrophage serum-free medium (Thermo Fisher Scientific, Waltham, MA, USA) supplemented with 10 mM (4-(2-hydroxyethyl)-1-piperazineethanesulfonic acid) HEPES and 5 μg/mL of Gentamycin) or 10% human AB serum (Geminin bioproducts, West Sacramento, CA, USA), respectively. IL-27-induced macrophages (iMac or Abi) were generated from the CD14+ monocytes by culturing for seven days in the presence of 25 ng/mL of M-CSF or 10% AB serum with 100 ng/mL of IL-27 (R&D Systems). The culture media was changed with fresh media every 3–4 days. All induced macrophages (Ab/Abi and mMac/iMac) cells were maintained in D-MEM medium (Thermo Fisher Scientific) containing 10% FBS (HyClone Laboratories, Logan, UT), 10 mM HEPES, and 5 μg/mL Gentamicin.

### 4.2. RNA Extraction, Library Preparation, and Sequencing

Total RNA from mMac/iMac (three donors: donor S1, S2, S3) and Ab/Abi (two donors: donor 90, 91) macrophages was extracted using TRIzol reagent (Thermo Fisher Scientific) following the manufacturer’s instructions. Contaminating genomic DNA was removed by DNAase treatment with a Turbo DNA-free Kit (Ambion Inc. Foster City, CA, USA). The integrity, concentration, and purity of total RNA were checked using a Nanodrop-1000 (NanoDrop Technologies, Wilmington, DE, USA) and Agilent 2100 Bioanalyzer (Agilent Technologies, Santa Clara, CA, USA). All samples had an RNA integrity number (RIN) ≥8.0 and an optical density 260/280 nm ratio ≥1.9.

Total RNA (2 μg) was subjected to ribosomal RNA (rRNA) depletion using a Ribo-Zero Gold rRNA Remove Kit (Illumina Inc., San Diego, CA, USA). The generation of RNA libraries for sequencing was made using the TruSeq Stranded mRNA Library Prep Kit (Illumina Inc.) following the company’s recommendations. The mRNA was captured with oligo-dT coated magnetic beads from 100 ng of total RNA, and then fragmented before a random-primed cDNA synthesis. The resulting double-strand cDNA was used as the input to a standard Illumina library prep with end-repair, adapter ligation, and PCR amplification. A 125-bp paired-end sequencing run was performed on the Illumina HiSeq2500 (San Diego, CA, USA) by the Center for Cancer Research Sequencing Facility (CCR-SF) (for ABI) and the Beijing Genomic Institute (BGI) (for IMAC). The sequencing data were submitted to the National Center for Biotechnology Information Sequence Read Archive under accession no. PRJNA559359.

### 4.3. Sequencing Data Processing

The sequence data from sequencing facilities were mapped to the human reference genome (hg38) using HISAT2 (v2.1.0) [71] with options --dta --rna-strandness RF for the stranded library. The transcripts from each sample were assembled using StringTie (v1.3.5) [72] with a stranded library parameter --rf. Constructed transcripts from all samples were merged by StringTie and annotated with Ensembl human gene annotation (release 93) [73] using GffCompare (v0.10.4) [74].

### 4.4. Novel lncRNA Prediction

Based on the assembly and GffCompare mapping results, un-annotated transcripts >200 bp with class code “i” (intronic), “u” (intergenic), and “x” (opposite strand exon) for protein-coding genes were retained. These transcripts were then assessed for their coding potential using the Coding-Potential Assessment Tool (CPAT v1.2.3) [75] and Coding-Non-Coding Index (CNCI v2) [76] with the recommended cut-off values (CPAT <0.364, CNCI <0). The transcripts were further compared with GENCODE lncRNA (release 29) [77], NONCODE2016 (v5.0) [78], and LNCipedia (v5.2) [79] using GffCompare (v0.10.4). Transcripts with class code “=” were classified as known lncRNAs while the rest were classified as novel.

### 4.5. Identification of lncRNA Targets

For the prediction of regulatory lncRNAs, genes were selected using Bedtools (v2.25.0) [80] from which lncRNAs were located 100 kbp upstream and downstream. LncRNA-mRNA interactions were predicted using LncTar (http://www.cuilab.cn/lnctar) [81] with an algorithm to find the minimum free energy joint structure by calculating the normalized binding free energy. In the present study, most of the lncRNA targets were from LncTar.

### 4.6. Differential mRNA and lncRNA Gene Expression Analysis

Reads in FASTQ format from the IMAC and ABI experiments were quantified at the transcript level using Salmon (v0.11.4) [82] against the Ensembl human gene (release 93) for mRNAs and against GENECODE lncRNA (release 29) plus un-annotated lncRNAs. The read count matrix was then aggregated to the gene level using tximport (v1.10.1) [83] and delivered to edgeR (v3.26.6) [39] in R (v3.5.3) [84] for the differential expression analysis, where a general linear model (GLM) was designed with donors + treatment for paired sample comparisons. The significant differential mRNAs/lncRNAs were selected based on fold change ≥2 or ≤−2 and false discovery rate (FDR) <0.05. A heat map was generated by pheatmap (v1.0.12) [85] using log_2_ fold change from log_2_ counts per million (logCPM) by edgeR.

### 4.7. Gene Ontology and Pathway Enrichment Analysis

Differentially expressed genes in both the IMAC and ABI experiments were enriched for Gene Ontology (GO) [86] and the Kyoto Encyclopedia of Genes and Genomes (KEGG) [87] pathways using DAVID [40]. FDR < 0.05 was considered as significantly enriched.

### 4.8. Co-Expression Network Analysis

Weighted gene co-expression network analysis (WGCNA) (v1.67) [88] in R (v3.5.3) with differentially expressed mRNAs and lncRNAs in both iMac and Abi compared to mMac and Ab, respectively, was used to construct and analyze a co-expression network. A topological overlap matrix (TOM) dissimilarity was used for generating a hierarchical clustering tree (dendrogram) of genes. Co-expressed genes were clustered into different modules by cutting branches of the tree using the dynamic tree cut algorithm [89]. GO and pathway enrichment analysis for each module were performed using STRING (v11.0) [90]. The differential genes in the top pathway of the module were visualized in Cytoscape (v3.7.1) [91]. A protein–protein interaction was from STRING. A lncRNA-mRNA interaction was from the prediction by Bedtools (v2.25.0) or LncTar.

### 4.9. Semi-Quantitative RT-PCR for lncRNA Validation

Cells from five donors (donors 1, 2, 3, 4, 5 were different to the donors for sequencing given the lack of peripheral blood mononuclear cell (PBMC) were washed with cold PBS twice and then RNA was extracted using the RNeasy Isolation Kit (QIAGEN, USA). Total cDNA was prepared using TaqMan reverse transcription reagents (Applied Biosystems, CA, USA). To quantitate the relative expression of the novel lncRNAs, quantitative real-time PCR was performed using the TaqMan universal PCR master mix (Thermo Fisher Scientific) following the manufacturer’s instructions on CFX96 Real-Time system (Bio-Rad Laboratories, CA, USA). Custom gene-specific primers and probes were made by Thermo Fisher Scientific. The relative expression of each lncRNA was calculated using the 2^−∆∆Ct^ (cycle threshold, Ct) method and normalized to GAPDH expression [35]. Statistical analysis (Student’s *t*-test with homogeneous variances) was undertaken using R.

### 4.10. Lnc RNA, Plamids, and Transfection

All selected lncRNAs (LHRI_LNC-2, 3, 5, and 6) were synthesized by Integrated DNA Technologies (Coralville, IA). These were digested and subcloned in the plasmid pcDNA3.1(+) (Thermo Fisher Scientific) to construct each expression plasmid. The empty vector was used as a control in the transfection experiments. The plasmids were transfected into HEK293T with TransIT-293 (Mirus Bio LLC, Madison, WI), according to the manufacturer’s protocol. The HEK293T cells were washed and infected with the HIV_luc_ virus.

### 4.11. Viruses and Infection

The HIV pseudotyped virus, HIV_luc_, was prepared as previously described [29]. LncRNA transfected HEK293T cells were infected with HIV_luc_ for 2 h. The infected cells were washed and then cultured for two days. Virus infection was monitored by luciferase activity using a Bright-Glo Luciferase Assay System (Promega, Madison, WI, USA).

## 5. Conclusions

Our study presents differentially expressed lncRNA and mRNA regulated by IL-27 in human macrophages. We found 2691 novel lncRNA-transcripts with 88 among them significantly regulated by IL-27. Furthermore, we found that these lncRNAs are involved in the immune system and cell cycle pathways. This suggests that lncRNA may play an important role in IL-27 antiviral or cellular function. Our findings contribute to a better understanding of IL-27 immune functions in macrophages and provide a resource for lncRNA research.

## Figures and Tables

**Table 1:** The number of long non-coding RNA (lncRNA) genes differentially expressed in Interleukin (IL)-27-induced macrophages.

Comparison	Upregulation		Downregulation	
	Known	Novel	Known	Novel
iMac vs. mMac	58	62	74	113
Abi vs. Ab	84	45	171	152

**Table 2:** Most significant Gene Ontology (GO) terms and Kyoto Encyclopedia of Genes and Genomes (KEGG) pathways in macrophages regulated by IL-27.

Category	Term	Count	Bonferroni Adjusted *p* Value
GO_BP	GO:0006955~immune response	38	1.69 × 10^−9^
GO_BP	GO:0002504~antigen processing and presentation of peptide or polysaccharide antigen via MHC class II	9	2.14 × 10^−6^
GO_BP	GO:0019886~antigen processing and presentation of exogenous peptide antigen via MHC class II	15	2.76 × 10^−5^
GO_BP	GO:0006954~inflammatory response	29	5.29 × 10^−5^
GO_BP	GO:0060333~interferon-gamma-mediated signaling pathway	12	8.26 × 10^−4^
GO_BP	GO:0031295~T cell costimulation	12	2.18 × 10^−3^
GO_BP	GO:0007067~mitotic nuclear division	20	5.58 × 10^−3^
GO_BP	GO:0019882~antigen processing and presentation	10	5.87 × 10^−3^
GO_BP	GO:0051301~cell division	24	7.09 x 10^−3^
GO_CC	GO:0030658~transport vesicle membrane	12	1.09 × 10^−7^
GO_CC	GO:0042613~MHC class II protein complex	10	1.33 × 10^−7^
GO_CC	GO:0071556~integral component of lumenal side of endoplasmic reticulum membrane	10	2.34 × 10^−6^
GO_CC	GO:0005886~plasma membrane	137	1.28 × 10^−5^
GO_CC	GO:0012507~ER to Golgi transport vesicle membrane	11	5.09 × 10^−5^
GO_CC	GO:0030666~endocytic vesicle membrane	12	5.63 × 10^−5^
GO_CC	GO:0030669~clathrin-coated endocytic vesicle membrane	10	6.50 × 10^−5^
GO_CC	GO:0005819~spindle	13	4.49 × 10^−3^
GO_CC	GO:0005615~extracellular space	54	5.76 × 10^−3^
GO_MF	GO:0042605~peptide antigen binding	10	2.35 × 10^−6^
GO_MF	GO:0032395~MHC class II receptor activity	8	6.17 × 10^−6^
KEGG	hsa05150:Staphylococcus aureus infection	19	3.72 × 10^−13^
KEGG	hsa04672:Intestinal immune network for IgA production	15	3.26 × 10^−9^
KEGG	hsa05330:Allograft rejection	13	3.14 × 10^−8^
KEGG	hsa05332:Graft-versus-host disease	12	1.34 × 10^−7^
KEGG	hsa04145:Phagosome	22	1.48 × 10^−7^
KEGG	hsa04940:Type I diabetes mellitus	12	2.38 × 10^−6^
KEGG	hsa05320:Autoimmune thyroid disease	13	2.43 × 10^−6^
KEGG	hsa05323:Rheumatoid arthritis	16	2.94 × 10^−6^
KEGG	hsa05310:Asthma	10	1.45 × 10^−5^
KEGG	hsa05322:Systemic lupus erythematosus	18	2.96 × 10^−5^
KEGG	hsa04612:Antigen processing and presentation	13	2.03 × 10^−4^
KEGG	hsa05321:Inflammatory bowel disease (IBD)	12	2.42 × 10^−4^
KEGG	hsa04110:Cell cycle	16	3.06 × 10^−4^
KEGG	hsa05166:HTLV-I infection	23	3.80 × 10^−4^
KEGG	hsa05416:Viral myocarditis	11	6.17 × 10^−4^
KEGG	hsa04514:Cell adhesion molecules (CAMs)	16	1.69 × 10^−3^
KEGG	hsa05140:Leishmaniasis	11	4.69 × 10^−3^
KEGG	hsa05168:Herpes simplex infection	17	9.15 × 10^−3^

**Table 3:** Most significant KEGG/Reactome pathways and GO terms associated with weighted gene correlation network analysis (WGCNA) modules.

Module	Term	mRNA Count	FDR
Brown	Reactome: Immune System	26	1.71 × 10^−6^
	KEGG: Staphylococcus aureus infection	8	7.16 × 10^−9^
	GO-BP: defense response	30	2.05 × 10^−13^
	GO-BP: immune system process	36	5.40 × 10^−11^
	GO-BP: positive regulation of immune system process	23	1.68 × 10^−10^
	GO-BP: immune response	29	1.78 × 10^−10^
	GO-BP: positive regulation of immune response	18	5.31 × 10^−9^
	GO-BP: regulation of immune system process	25	1.70 × 10^−8^
	GO-BP: positive regulation of response to stimulus	30	1.70 × 10^−8^
	GO-BP: regulation of immune response	20	3.07 × 10^−8^
	GO-BP: innate immune response	16	1.74 × 10^−6^
	GO-BP: response to stress	34	2.46 × 10^−6^
	GO-BP: regulation of response to external stimulus	16	4.25 × 10^−6^
	GO-BP: cell surface receptor signaling pathway	27	4.90 × 10^−6^
	GO-BP: activation of immune response	12	7.96 × 10^−6^
	GO-BP: inflammatory response	13	7.96 × 10^−6^
Yellow	Reactome: Cell Cycle, Mitotic	31	4.54 × 10^−26^
	Reactome: Cell Cycle	32	2.93 × 10^−25^
	KEGG: Cell cycle	13	8.73 × 10^−13^
	GO-BP: mitotic cell cycle	38	1.55 × 10^−31^
	GO-BP: cell cycle	47	1.55 × 10^−31^
Blue	Reactome: Regulation of Complement cascade	6	2.50 × 10^−4^
	KEGG: Complement and coagulation cascades	6	1.60 × 10^−3^
	GO-BP: complement activation	6	2.40 x 10^−3^
Turquoise	Reactome: Immune System	25	3.20 × 10^−3^
	KEGG: Staphylococcus aureus infection	6	5.11 × 10^−5^
	GO-BP: immune response	26	1.40 × 10^−4^
	GO-BP: innate immune response	16	4.60 × 10^−4^

## Supplementary Materials

Supplementary materials can be found at https://www.mdpi.com/1422-0067/20/24/6207/s1.

## Abbreviations

ABI	IL-27 treated human AB-serum induced macrophages
APCs	Antigen presenting cells
EBI3	Epstein-Barr virus-induced gene 3
GO	Gene Ontology
HEPES	4-(2-hydroxyethyl)-1-piperazineethanesulfonic acid
HIV	Human immunodeficiency virus (HIV)
HBV	Hepatitis B virus
HSV-1	Herpes simplex virus type 1
IMAC	IL-27-treated M-CSF induced macrophages
JAK/STAT	Janus kinase/signal transducer and activator of transcription
KEGG	Kyoto Encyclopedia of Genes and Genomes
lncRNAs	Long non-coding RNAs
logCPM	Log2 counts per million
MAPKs	Mitogen-activated protein kinases
M-CSF	Macrophage colony-stimulating factor
MDMs	Monocyte-Derived Macrophages
MHC	Major histocompatibility complex
PBMCs	Peripheral blood mononuclear cells
RT-qPCR	Quantitative reverse transcription polymerase chain reaction
TLR	Toll like receptor
WGCNA	Weighted gene correlation network analysis
