# Correction: Hu et al. Profiles of Long Non-Coding RNAs and mRNA Expression in Human Macrophages Regulated by Interleukin-27. *Int. J. Mol. Sci.* 2019, *20*, 6207

**DOI:** 10.3390/ijms22105129

**Published:** 2021-05-12

**Authors:** Xiaojun Hu, Suranjana Goswami, Ju Qiu, Qian Chen, Sylvain Laverdure, Brad T. Sherman, Tomozumi Imamichi

**Affiliations:** Laboratory of Human Retrovirology and Immunoinformatics, Frederick National Laboratory for Cancer Research, Frederick, MD 21702, USA; xiaojun.hu@usda.gov (X.H.); suranjana.goswami@nih.gov (S.G.); ju.qiu@nih.gov (J.Q.); chenq3@mail.nih.gov (Q.C.); sylvain.laverdure@nih.gov (S.L.); bsherman@mail.nih.gov (B.T.S.)

The authors wish to make the following corrections to this paper [1]:

We have recently been made aware by Danielle and Jean Thierry-Mieg (NCBI. NLM. NIH) of some errors and omissions in the Results section of our recent paper. The first paragraph of said Results currently reads as follows:

Finally, 2691 candidate lncRNA transcripts were obtained (Table S2). Among them, 53% were from intergenic regions, 27% from intronic regions, and 20% were antisense to protein-coding loci.

To set straight the scientific record, we would like to make the following corrections:

After removing ribosomal RNAs, 2613 candidate lncRNA transcripts were obtained (Table S2). Among them, 51% were from intergenic regions, 28% from intronic regions, and 21% were antisense to protein-coding loci. 

The authors declare that this change does not affect the validity of the conclusion. The authors would like to apologize for any inconvenience caused to the readers by these changes.

## Data Availability

The data presented in this study are openly available in the NCBI SRA accession no. PRJNA559359.

## Supplementary Materials

The following are available online at https://www.mdpi.com/article/10.3390/ijms22105129/s1.
