# Supplementary material for: Profiles of Long Non-Coding RNAs and mRNA Expression in Human Macrophages Regulated by Interleukin-27
Source: Int J Mol Sci. 2019 Dec 9;20(24):6207. doi: 10.3390/ijms20246207 (PMC6941108; doi:10.3390/ijms20246207)
Supplement: Supplementary file 1 [file ijms-20-06207-s001.zip › ijms-663677-supplementary/ijms-663677-supplementary.pdf]

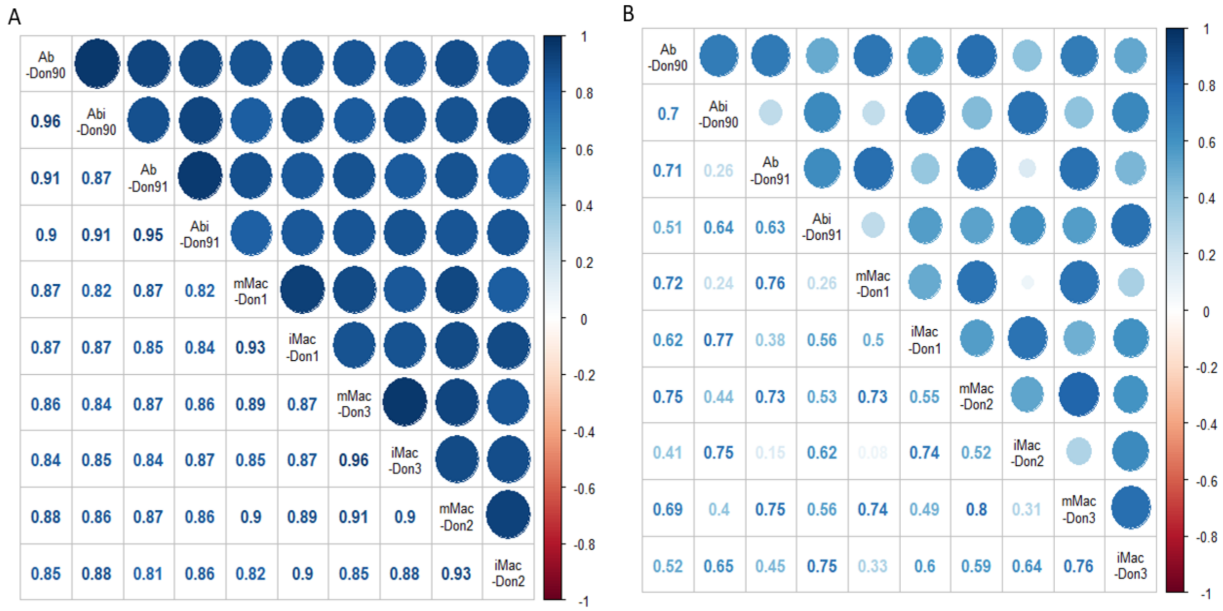

Supplementary Figure S1. Sample paired-wise correlations. A. using all genes. B. using 434 significant genes. The numbers are Pearson correlation coefficients. Circle size and color indicate correlation levels.

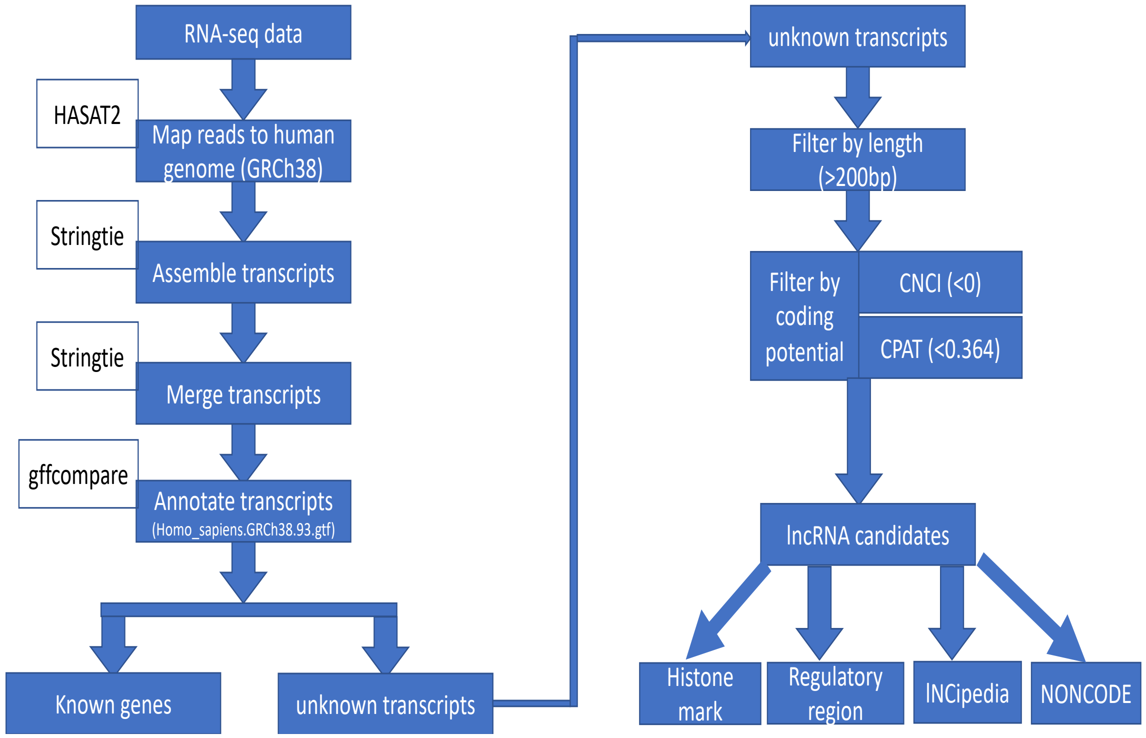

Supplementary Figure S2. Pipeline for selecting lncRNAs. A schematic overview of the pipeline employed for selecting candidate lncRNAs.

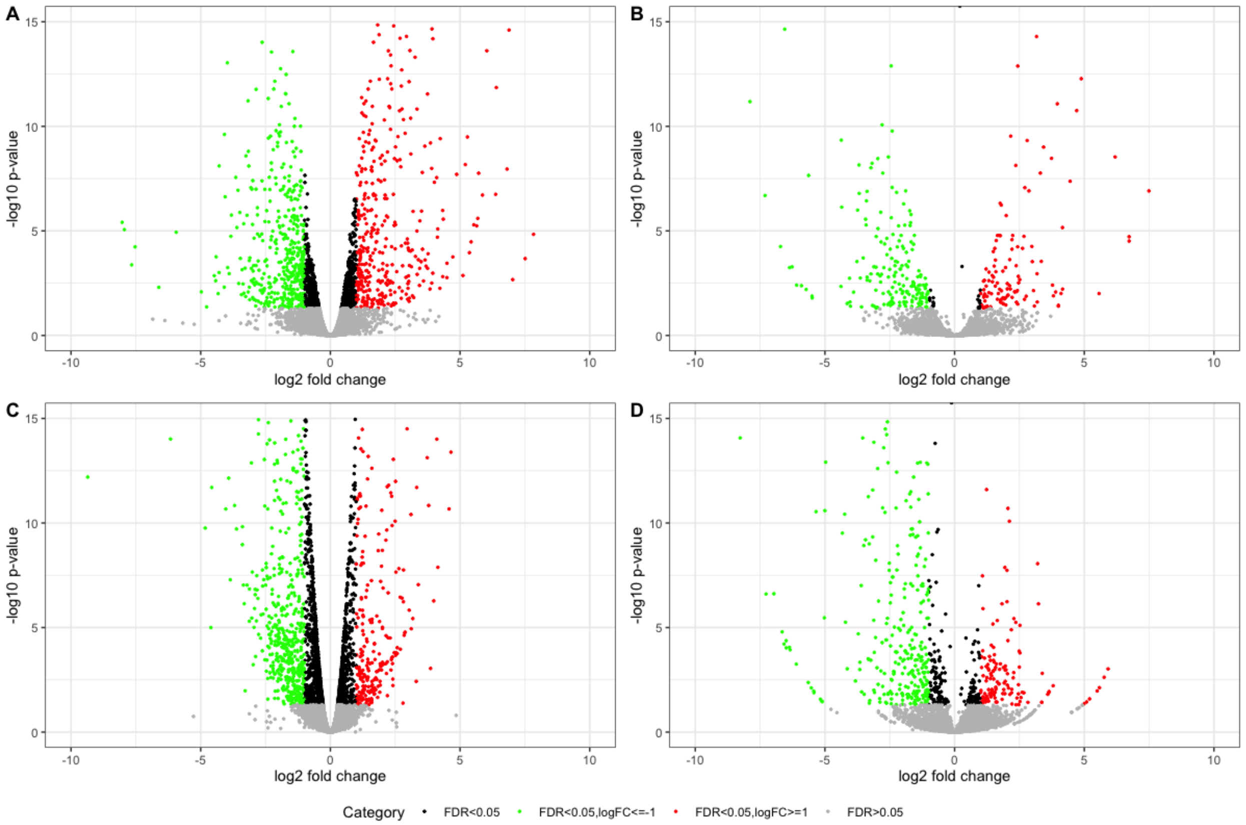

Supplementary Figure S3. Volcano plots of mRNAs and lncRNAs. The values on the X axes are the log2 fold change. The values on the Y axes are the -log10(false discovery rate (FDR)). The red point represents differentially up-regulated ones with filtering criteria fold change >= 2 and p-value < 0.05. The green point represents differentially down-regulated ones with filtering criteria fold change <= -2 and p-value < 0.05. (A) messenger RNAs (mRNAs) in IMAC. (B). lncRNAs in IMAC. (C) mRNAs in ABI. (D). lncRNAs in ABI.

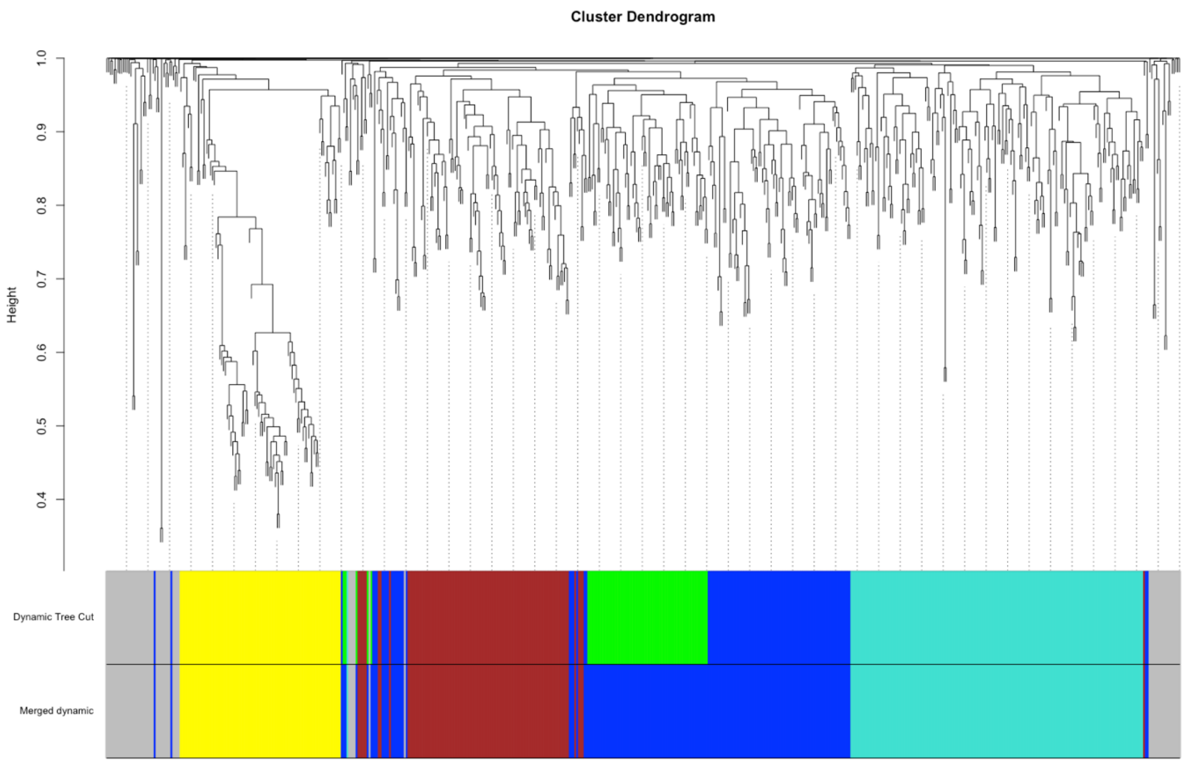

Supplementary Figure S4. Visualization of WGCNA module detection. Gene clustering tree (dendrogram) obtained by hierarchical clustering of TOM-based dissimilarity of 434 mRNAs and 146 lncRNAs. The color rows below the dendrogram indicate module membership identified by the two methods: dynamic tree cut and merged dynamic branch cutting (used in the further analysis).

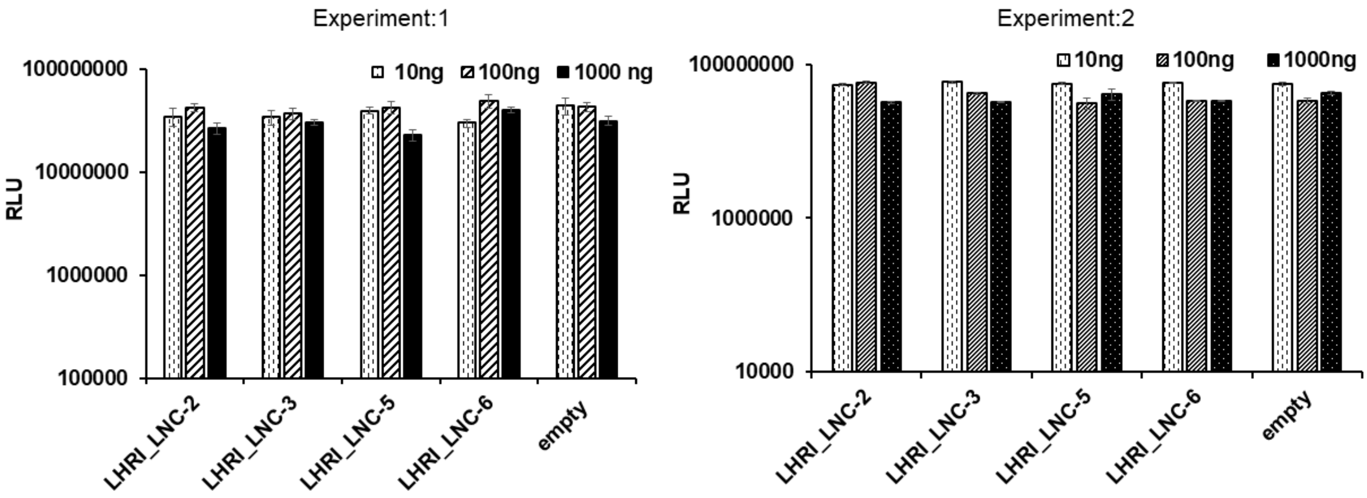

Supplementary Figure S5. HEK293T were treated with 10, 100, 1000ng of plasmid (LHRI_LNC-2,3,5,6) and then infected with HIV_{luc} and cultured for 48hrs. Luciferase activity is shown in relative units (RLU). This was repeated twice (Experiment1 and 2) to verify the results. No antiviral effect was observed.
